# Effects of vaccination against paratuberculosis on tuberculosis in goats: diagnostic interferences and cross-protection

**DOI:** 10.1186/1746-6148-8-191

**Published:** 2012-10-16

**Authors:** Bernat Pérez de Val, Miquel Nofrarías, Sergio López-Soria, Joseba M Garrido, H Martin Vordermeier, Bernardo Villarreal-Ramos, Maite Martín, Eugenia Puentes, Ramón A Juste, Mariano Domingo

**Affiliations:** 1 Centre de Recerca en Sanitat Animal (CReSA), UAB-IRTA, Campus de la Universitat Autònoma de Barcelona, Edifici CReSA, 08193, Bellaterra, Catalonia, Spain; 2 Department of Animal Health, NEIKER-Tecnalia, 48160, Derio, Bizkaia, Spain; 3 TB Research Group, Animal Health and Veterinary Laboratories Agency (AHVLA)-Weybridge, New Haw, Addlestone, Surrey, KT15 3NB, United Kingdom; 4 CZ Veterinaria S.A., 36400, Porriño, Pontevedra, Spain; 5 Departament de Sanitat i Anatomia Animals, Universitat Autònoma de Barcelona, 08193, Bellaterra, Catalonia, Spain

**Keywords:** Tuberculosis, Paratuberculosis, Goat, Vaccine, Diagnostic, Interferon gamma

## Abstract

**Background:**

Most countries carrying out campaigns of bovine tuberculosis (TB) eradication impose a ban on the use of mycobacterial vaccines in cattle. However, vaccination against paratuberculosis (PTB) in goats is often allowed even when its effect on TB diagnosis has not been fully evaluated. To address this issue, goat kids previously vaccinated against PTB were experimentally infected with TB.

**Results:**

Evaluation of interferon-γ (IFN-γ) secretion induced by avian and bovine tuberculins (PPD) showed a predominant avian PPD-biased response in the vaccinated group from week 4 post-vaccination onward. Although 60% of the animals were bovine reactors at week 14, avian PPD-biased responses returned at week 16. After challenge with *M. caprae*, the IFN-γ responses radically changed to show predominant bovine PPD-biased responses from week 18 onward. In addition, cross-reactions with bovine PPD that had been observed in the vaccinated group at week 14 were reduced when using the *M. tuberculosis* complex-specific antigens ESAT-6/CFP-10 and Rv3615c as new DIVA (differentiation of infected and vaccinated animals) reagents, which further maintained sensitivity post-challenge. Ninety percent of the animals reacted positively to the tuberculin cervical comparative intradermal test performed at 12 weeks post-infection. Furthermore, post-mortem analysis showed reductions in tuberculous lesions and bacterial burden in some vaccinated animals, particularly expressed in terms of the degree of extrapulmonary dissemination of TB infection.

**Conclusions:**

Our results suggest a degree of interference of PTB vaccination with current TB diagnostics that can be fully mitigated when using new DIVA reagents. A partial protective effect associated with vaccination was also observed in some vaccinated animals.

## Background

Caprine tuberculosis (TB), caused either by *Mycobacterium bovis* or *M. caprae*, and paratuberculosis (PTB), caused by *M. avium* subsp. *paratuberculosis* (*Map*), are endemic diseases in goat herds of the Iberian Peninsula [1-3]. Both infections may have an impact in terms of economic loss. Moreover, *M. caprae* and *Map* can be transmitted between domestic hosts and wildlife species [2,4]. In addition, *M. caprae* is a zoonotic agent [5-7] and *Map* has been associated with Crohn’s disease [8,9].

Control of PTB in small ruminants can be facilitated by vaccination because the bacterial burden is greatly reduced, containing the spread of the disease and preventing clinical expression [10]. Furthermore, vaccination against PTB with *Map*-killed formulations has been demonstrated to be an economically efficient strategy to achieve control of the disease in small ruminants [11-13] and cattle [14].

On the other hand, it has been shown that exposure to *M. avium* can interfere with the diagnosis of *M. tuberculosis* complex organisms such as *M. bovis or M. caprae*[15-17]. Similarly, there are some reports showing that natural infection with *Map* compromises TB diagnosis in cattle [18,19] and goats [20]. Therefore, PTB could also affect the specificity of diagnostic tests used in TB control programs. The Spanish eradication program of bovine TB expressly forbids the use of *Map*-based vaccines in cattle because of this potential interference with TB diagnostics. In contrast, vaccination against PTB in goats is permitted with the exception of a minority of herds that are subjected to TB control in some regions. To date, the potential effect of *Map*-based vaccines on the diagnosis of TB has only been investigated *post hoc* under field conditions [21,22].

It has been reported that TB diagnostic tests currently used in cattle, such as the skin test or the interferon-gamma (IFN-γ) assay, can be used for diagnosis of TB in goats [23-25]. Moreover, in recent years, new IFN-γ assays based on antigens secreted by active growing bacilli, such as the peptide cocktail ESAT-6/CFP-10 (E/C) or Rv3615c [26,27], have been developed as alternatives to bovine and avian tuberculins. These antigens are not present in either *M. bovis* BCG or *Map* and can be viewed as novel DIVA (differentiation of infected and vaccinated animals) regents able to distinguish TB-infected from TB- or PTB-vaccinated animals.

The aim of the present work was to assess how vaccination against *Map* affects standard and novel diagnostic tests in goats. Thus, we evaluated the interference of a commercial *Map*-killed vaccine on TB diagnosis in goats vaccinated and subsequently challenged with *M. caprae*. The objectives of the present work were: (1) to investigate the possible interference of vaccination before and after *M. caprae* infection on TB diagnostic tests (single and comparative skin tests and IFN-γ assay); (2) to assess the usefulness of DIVA-peptide candidates E/C and Rv3615c; and (3) to evaluate immunological and post-mortem indicators of the effects of PTB vaccination on *M. caprae* infection.

## Results

### Assessment of diagnostic tests

The effect of *Map* vaccination and subsequent infection with *M. caprae* on TB diagnostic assays based on cell-mediated immunity was assessed during this study. Four vaccinated goats were subjected to the tuberculin cervical comparative intradermal test (CIT) at week 14, and all were negative for TB and correctly classified as avian reactors. However, the four goats were classified as positive for TB if only the bovine tuberculin (PPD-B) result (as a single intradermal tuberculin test [SIT]) was considered (data not shown), which indicated that the specificity of the SIT was severely compromised by PTB vaccination. The skin test was repeated in all goats at week 26 (12 weeks post-infection [wpi] with *M. caprae*). In the vaccinated group, 9 of 10 goats were bovine reactors (positive for TB) using the CIT (1 goat was an avian reactor), but all goats were classified as TB reactors by applying the SIT interpretation. All unvaccinated goats were positive for TB with both tests (Table 1). Differences between groups in the mean specific thickness increase for each tuberculin were statistically significant only for ΔPPD-A (avian tuberculin), with a mean increase of 14.4 mm (11.9–17, 95% CI) in the vaccinated group versus a mean increase of 10.8 mm (8.8–12.9, 95% CI) in the control group (*p* < 0.05) (Table 1). Differences were also found in the mean value of ΔPPD-B minus ΔPPD-A. This value was significantly lower in the vaccinated group (5.2 mm, 2.9–7.5, 95% CI) than in the unvaccinated group (10.3 mm, 8.8–11.9, 95% CI) (*p* < 0.005) (Table 1).

**Table 1:** Increases in skin-fold thickness and corresponding results of tuberculin skin tests

**Group**	**Goat**	**ΔAv**^**a**^	**ΔBov**^**b**^	**ΔBov-ΔAv**	**CIT**	**SIT**
Vaccinated	1	14.2	22.6	8.4	+	+
	2	15.2	26.0	10.8	+	+
	3	17.6	20.9	3.3	+	+
	4	10.6	19.5	8.9	+	+
	5	16.2	18.1	1.9	+	+
	6	14.5	16.5	2.0	+	+
	7	6.2	14.2	8.0	+	+
	8	14.6	17.4	2.8	+	+
	9	13.2	19.4	6.2	+	+
	10	22.0	21.7	−0.3	- ^e^	+
	Mean (95% CI)	14.4 (11.9-17)*^f^	19.6 (17.5-21.7)	5.2 (2.9-7.5)**		
Unvaccinated	11	11.3	19.7	8.4	+	+
	12	15.0	24.1	9.1	+	+
	13	3.3	14.5	11.2	+	+
	14	9.8	18.3	8.5	+	+
	15	11.3	21.2	9.9	+	+
	16	8.7	24.6	16.0	+	+
	17	11.4	19.1	7.7	+	+
	18	10.1	22.5	12.4	+	+
	19	14.6	25.8	11.2	+	+
	20	12.7	21.6	8.9	+	+
	Mean (95% CI)	10.8 (8.8-12.9)	21.1 (19–23.2)	10.3 (8.8-11.9)		

Test results are scored as (−) negative or (+) positive.

^a^ ΔAv, increase in skin-fold thickness (mm) 72 h after PPD-A application.

^b^ ΔBov, increase in skin-fold thickness (mm) 72 h after PPD-B application.

^c^ CIT, Cervical comparative intradermal test.

^d^ SIT, Single intradermal test.

^e^ Avian reactor.

^f^ *p < 0.05; **p < 0.005 (unpaired two-sample Student’s *t*-test).

**Figure 1:**
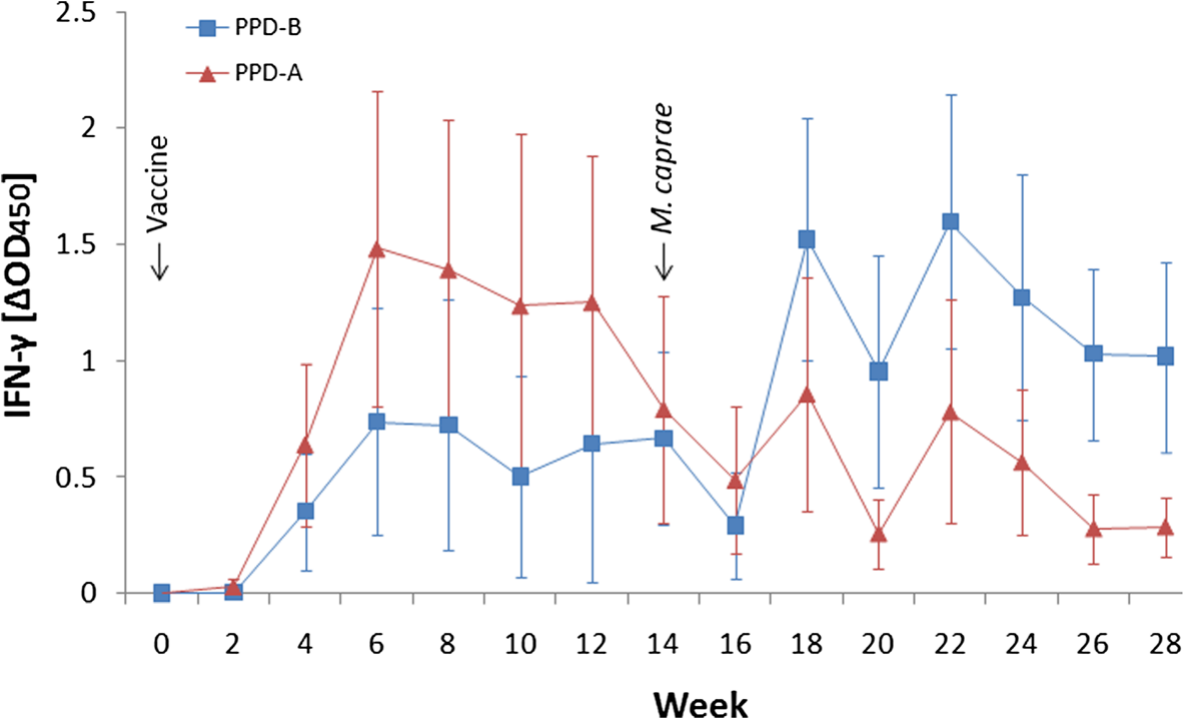
**Kinetics of IFN-γ released after stimulation of whole blood with tuberculins in the vaccinated group. **Avian tuberculin (PPD-A, ▲), bovine tuberculin (PPD-B, ■). Results are expressed as mean ΔOD_450_ (mean of OD_450_ of antigen-stimulated values with unstimulated values subtracted) ± 95% confidence interval.

The tuberculin-based IFN-γ test was performed every 2 weeks throughout the experiment. The kinetics of IFN-γ responses of the vaccinated group showed a PPD-A-biased response, which switched to a PPD-B-biased mean response 4 weeks after *M. caprae* infection (at week 18 of the experiment) (Figure 1). In the unvaccinated control group, a PPD-B-biased response was obtained 4 weeks after *M. caprae* infection, whereas no response was observed before this time point in any of the goats (data not shown). When the results were analyzed individually (Table 2), *Map*-vaccinated goats began showing responses to PPD-A (“avian reactors”) at week 2 after vaccination (2 of 10 goats), and all of them subsequently became avian reactors between weeks 4 and 10. However, 1 of 10 goats became a “bovine reactor” (positive to PPD-B) at week 12 of the experiment (12 weeks post-vaccination [wpv]), and this figure rose to 6 of 10 at week 14, in the blood samples taken just before infection with *M. caprae*. Interestingly, in the next blood sampling at 2 wpi (week 16), again 8 of 10 goats showed a stronger reaction to PPD-A than to PPD-B, and the 2 other vaccinated goats were negative to both tuberculins. By contrast, at week 18 (4 weeks after *M. caprae* challenge), 9 of 10 (90%) of the vaccinated goats were bovine reactors. At week 20, all goats were positive to PPD-B (bovine reactors), and these results were maintained during the rest of the trial with the exception of one goat that tested bovine-negative at week 24 and another that was an avian reactor at week 28 (see Table 2). In the unvaccinated group, all goats were bovine reactors at week 18 and remained so until the end of the experiment, with the exception of two goats that were negative at week 24 but returned to positivity at weeks 26 and 28 (data not shown).

**Table 2:** Results of the standard IFN-γ assay (Bovigam™)

**Goat**		**Week**													
	**0**	**2**	**4**	**6**	**8**	**10**	**12**	**14**	**16**	**18**	**20**	**22**	**24**	**26**	**28**
1	N	N	Av	Av	Av	Av	Av	Av	Av	Bov	Bov	Bov	Bov	Bov	Bov
2	N	Av	Av	Av	Av	Av	Av	Av	Av	Bov	Bov	Bov	Bov	Bov	Bov
3	N	N	Av	Av	Av	Av	Av	Bov	Av	Bov	Bov	Bov	Bov	Bov	Bov
4	N	N	Av	Av	Av	Av	Bov	Bov	Av	Bov	Bov	Bov	N	Bov	Bov
5	N	N	Av	Av	Av	Av	Av	Bov	N	Bov	Bov	Bov	Bov	Bov	Bov
6	N	N	Av	Av	Av	Av	Av	Av	Av	Bov	Bov	Bov	Bov	Bov	Bov
7	N	N	Av	Av	Av	Av	Av	Bov	N	Bov	Bov	Bov	Bov	Bov	Av
8	N	N	Av	Av	Av	Av	Av	Av	Av	Av	Bov	Bov	Bov	Bov	Bov
9	N	Av	Av	Av	Av	Av	Av	Bov	Av	Bov	Bov	Bov	Bov	Bov	Bov
10	N	N	Av	Av	Av	Av	Av	Bov	Av	Bov	Bov	Bov	Bov	Bov	Bov

Test results are classified as: Av, positive response to PPD-A; Bov, positive response to PPD-B; or N, negative.

Next, we assessed the interference of *Map* vaccination with the IFN-γ assay by employing the defined antigen reagents E/C and Rv3615c. Responses were assessed at the same five time points described in the Materials and Methods section. At weeks 14 (0 wpi) and 16, all goats were defined as TB-negative (ΔOD_450_ ≤ 0.05) according to the test results. At weeks 20, 24, and 28, 90%, 100%, and 70% of vaccinated goats were positive with the E/C cocktail and 100%, 90%, and 80% were positive with Rv3615c, respectively (Table 3). However, these proportions increased to 100%, 100%, and 90% at weeks 20, 24, and 28, respectively, when the E/C and Rv3615c results were considered together. Very similar results were obtained in the unvaccinated group, in which 100%, 80%, and 90% of goats were positive to E/C at weeks 20, 24, and 28, respectively; 100%, 80%, and 80% of goats were positive to Rv3615c; and 100%, 80%, and 90% of goats were positive again when combining the results for both antigens at the same time points (data not shown).

**Table 3:** Results of the IFN-γ assay using two DIVA reagents: ESAT-6/CFP10 (E/C) and Rv3615c

**Goat**	**Week**									
	**14**		**16**		**20**		**24**		**28**	
	**E/C**	**Rv3615c**	**E/C**	**Rv3615c**	**E/C**	**Rv3615c**	**E/C**	**Rv3615c**	**E/C**	**Rv3615c**
1	-	-	-	-	+	+	+	-	+	-
2	-	-	-	-	+	+	+	+	+	+
3	-	-	-	-	+	+	+	+	+	+
4	-	-	-	-	+	+	+	+	+	+
5	-	-	-	-	+	+	+	+	+	+
6	-	-	-	-	-	+	+	+	-	+
7	-	-	-	-	+	+	+	+	-	+
8	-	-	-	-	+	+	+	+	-	-
9	-	-	-	-	+	+	+	+	+	+
10	-	-	-	-	+	+	+	+	+	+

Test results are classified as (−) negative or (+) positive.

Seropositivity to *Map* was assessed using the Paratub.Serum-S ELISA kit (Institut Pourquier, Montpellier, France) applying the cut-off defined above. All goats (n = 20) were negative at week 0. Unvaccinated goats also remained negative during the experiment (data not shown). In contrast, 1 of 10 (10%) and 5 of 10 (50%) vaccinated goats were positive or doubtful at weeks 14 (0 wpi) and 28 (14 wpi), respectively, with different degrees of intensity (Table 4). The mean intensity of antibody response (%S/P) increased moderately, but was statistically significant (*p* < 0.05), from 20% (4%–36%, 95% CI) at week 14 to 55% (19%–91%, 95% CI) at week 28 (2 weeks after CIT).

**Table 4:** **Seroreactivity to *Map *(Paratub.Serum-S™ ELISA)**

**Goat**	**Week**					
	**0**		**14**		**28**^**a**^	
	**Result**	**S/P**^**b**^	**Result**	**S/P**	**Result**	**S/P**
1	-	0%	-	30%	+	130%
2	-	0%	-	0%	-	0%
3	-	1%	-	8%	d	51%
4	-	0%	-	3%	+	113%
5	-	0%	-	36%	+	56%
6	-	0%	-	16%	-	19%
7	-	0%	-	6%	-	4%
8	-	0%	+	86%	+	157%
9	-	0%	-	4%	-	9%
10	-	0%	-	11%	-	11%

Test results are classified as (−) negative, (+) positive, or (d) doubtful.

^a^ Two weeks after CIT.

^b^ S/P, Seroreactivity rates.

### Pathology and bacteriology

At necropsy on week 28 (14 wpi), visible pathology typical of TB was observed in the lungs and pulmonary lymph nodes (LN) of all animals irrespective of their vaccination status. Of the seven lung lobes evaluated, the vaccinated group presented an average of three lobes with gross lesions (2–4, 95% CI), slightly lower than the mean of four lobes (3–5, 95% CI) found in the unvaccinated group, but this difference was not statistically significant (*p* = 0.324).

**Figure 2:**
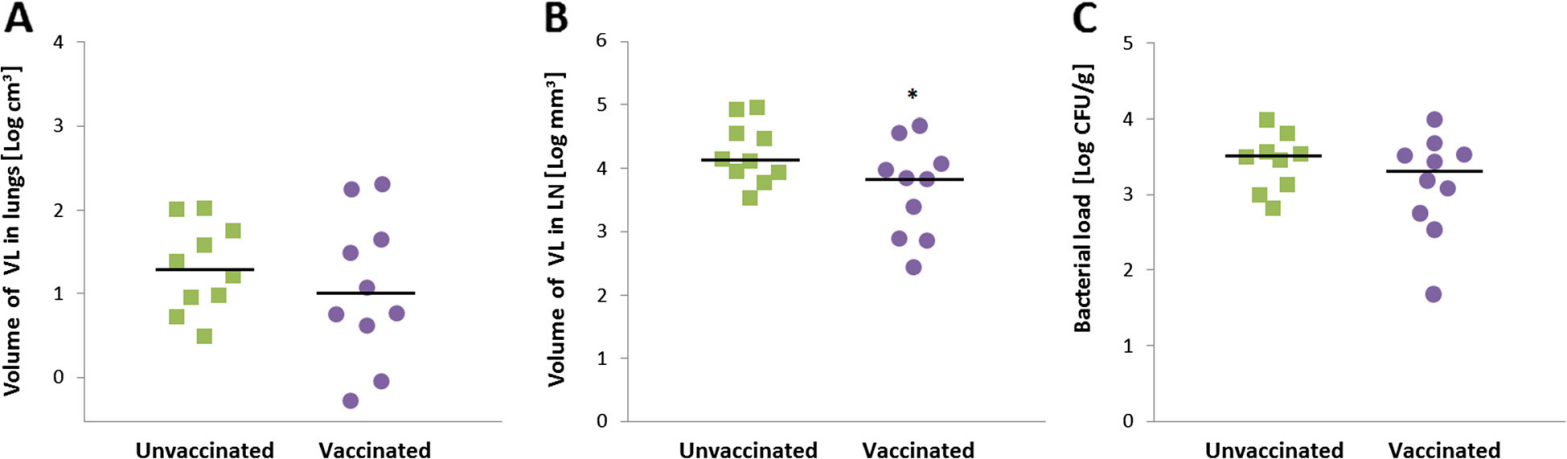
**Post-mortem analysis as measured by gross pathology and bacterial burden. **Results are plotted for individual goats. (**A**) Total volume of visible lesions (VL) in lungs as log_10_ cm^3^. (**B**) Total volume of VL in respiratory lymph nodes (LN) as log_10_ mm^3^. (**C**) Bacterial load as log_10_ cfu/g. (●) vaccinated goats, (■) unvaccinated goats. Horizontal lines indicate median values. Significance determined by unpaired *t*-test: *p < 0.05.

The mean log_10_-transformed volume of gross lesions in lungs was similar in the unvaccinated (1.3 log_10_ cm^3^; 1–1.7, 95% CI) and vaccinated (1.1 log_10_ cm^3^; 0.5–1.6, 95% CI) groups (*p* = 0.470), but values were spread over a wider range in the vaccinated group. By contrast, statistically significant differences between vaccinated and control goats were found after assessing the gross pathology in LN: The unvaccinated animals presented with a mean log_10_-transformed volume of visibly affected tissue of 3.9 log_10_ mm^3^ (3.6–4.3, 95% CI) compared with 3.3 log_10_ mm^3^ (2.7–3.8, 95% CI) obtained in the vaccinated group (*p* < 0.05). The individual pathological parameters are represented in Figure 2A and B.

In addition, none of the vaccinated goats showed extrapulmonary gross lesions, whereas dissemination was observed in 4 of 10 unvaccinated goats that presented with gross lesions beyond the thoracic area (2 goats with lesions in retropharyngeal LN, 2 goats with lesions in mesenteric LN, and 1 goat with lesions in the spleen). These lesions were histopathologically confirmed to be tuberculous.

In three animals, focal lesions were also found in the LN draining the *Map* vaccine inoculation point (axillary, prescapular, or subcutaneous LN). These lesions were attributed to the vaccine inoculation because of their localization and histological characteristics: lesions predominantly necrotizing rather than granulomatous, without Langerhans cells, but surrounded by dispersed polymorphonuclear cells and abundant fibrosis.

Finally, the mycobacterial load in LN was calculated as log_10_ cfu/g. The bacterial burden in the pulmonary and retropharyngeal LN of all animals (n = 20) ranged from 2 to 4 log_10_ cfu/g (Figure 2C). The total bacterial load in the unvaccinated group was 3.5 log_10_ cfu/g (3.2–3.7, 95% CI), slightly higher than that in the vaccinated group (3.1 log_10_ cfu/g, 2.7–3.6, 95% CI). However, this difference was not statistically significant (*p* = 0.203).

### Cross-sectional analysis

**Figure 3:**
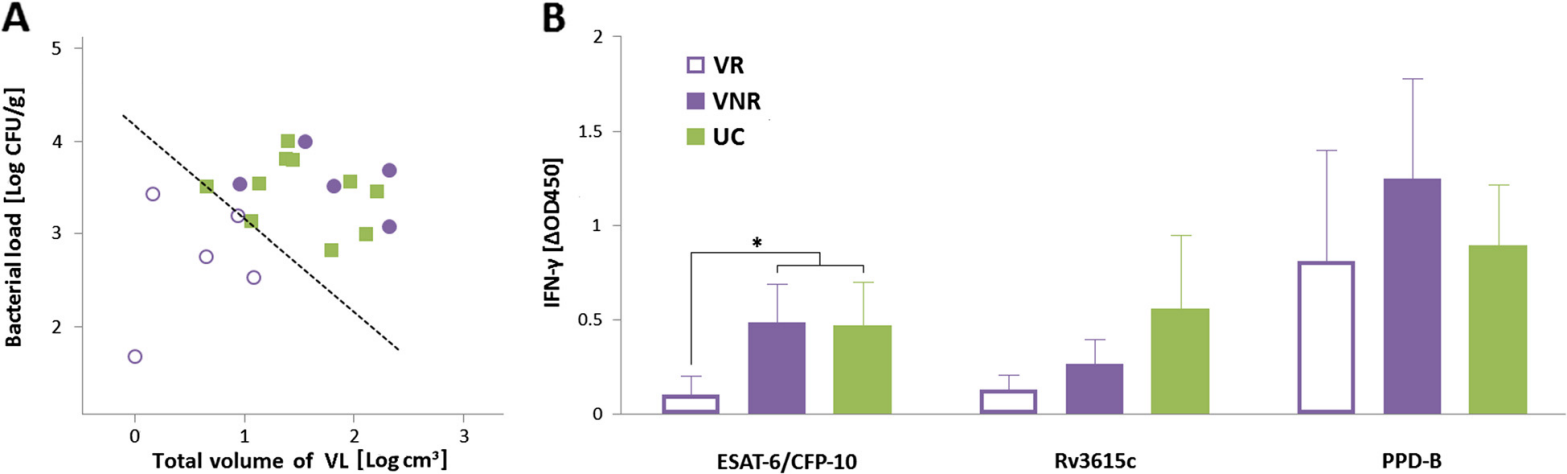
**Protective efficacy measured by post-mortem results, and cross-sectional comparison of IFN-γ results with vaccination outcome. **(**A**) Correlation between pathological and bacteriological parameters of all goats (n = 20). Dashed line (y = −x + 4.16) indicates the threshold defined to divide vaccine responders (VR) and vaccine non-responders (VNR). Symbols represent vaccine cross-protection outcome: (■) unvaccinated controls (UC), (○) VR goats, (●) VNR goats. (**B**) Specific IFN-γ responses at week 28 of the experiment as determined by ELISA after stimulation of whole blood with PPD-B, ESAT-6/CFP-10, or Rv3615c. Results are represented in relation to vaccine cross-protective outcome and are expressed as mean ΔOD_450_ ± 95% confidence interval. Significances determined by non-parametric Kruskal-Wallis test: *p < 0.05.

To evaluate the vaccine effect in terms of heterologous protection, post-mortem parameters were also assessed in combination. A mild positive correlation between bacteriological and pathological findings was found by correlating individual logarithmic values of bacterial load and total volume of visible lesions (r = 0.386, *p* < 0.05). However, a higher dispersion of individual values was observed in the vaccinated group compared with the unvaccinated group (Figure 3A), and a stronger positive correlation was found when only vaccinated goats were assessed (r = 0.523, *p* < 0.05), whereas results obtained from unvaccinated goats did not show significant correlations (r = -0.274, *p* > 0.05).

To further analyze the reductions in post-mortem parameters associated with vaccination, a cross-sectional study was performed on the basis of the criteria described in a previous TB vaccine efficacy trial [28]. The vaccinated animals were divided into two subgroups according to the severity of the pathology and the bacterial burden. This division was established by combining the data of the total volume of gross lesions and the bacterial load in LN. The mean between the volume of lesions (log_10_ cm^3^) and the bacterial load (log_10_ cfu) was thereby calculated for each animal, and the cut-off point to classify the animals into categories of distinct vaccination outcomes was defined as the minimum value obtained among the unvaccinated control animals (Figure 3A). Subsequently, a “vaccine responders” (VR) subgroup of five goats and another “vaccine non-responders” (VNR) subgroup of five goats were defined (Figure 3A, open and closed circles).

Once the subgroup division was applied as described in the previous paragraph, the assumed pathological and bacteriological differences between the new subgroups were checked. The total volume of visible lesions and the bacterial load were significantly lower in VR compared with VNR and unvaccinated control (UC) animals (*p* < 0.05).

The release of IFN-γ specific to E/C, Rv3615c, and PPD-B at week 28 (performed just before sacrifice of the animals) was also compared between the VR, VNR, and UC groups (Figure 3B). A significantly lower IFN-γ to E/C response was found in VR animals than in VNR and UC animals (*p* < 0.05). The relationship between E/C-specific IFN-γ response and disease severity was confirmed by finding a positive correlation of IFN-γ-specific secretion to E/C at week 28 with the total volume of gross lesions (Spearman rank = 0.546, *p* < 0.01) and the bacterial load (but at the statistical limit of significance [Spearman rank = 0.379, *p* = 0.050], data not shown).

## Discussion and conclusions

The present experimental trial in goats was designed with the aim to determine the effects of *Map* vaccination on current TB diagnostic tests and assess the effects of PTB vaccination on TB infection.

All experimental goats became infected irrespective of vaccination status. However, a reduction in pulmonary pathology was observed in some vaccinated individuals compared with the unvaccinated group. Another remarkable finding was that all vaccinated goats showed only TB lesions at the site of infection (i.e., lungs and associated LN) in contrast to the increased dissemination frequency in non-vaccinated animals. This finding is analogous to the results of subcutaneous vaccination against PTB with the *Map*-killed vaccine in cattle subsequently challenged with *Map*. The vaccine in those studies induced systemic immunity, preventing bacteremia and, consequently, dissemination of mycobacteria from the primary infection site. However, they did not prevent the establishment of the initial infection [10]. Similar results were obtained in another experimental infection with *M. caprae* in which three of six (50%) infected goats showed gross TB lesions in mesenteric LN [29]. This outcome is also consistent with findings observed in field cases of TB in goats [30-32].

In this sense, the present results indicate a certain degree of containment of dissemination of the infection from the primary complex, even when a significant reduction in the bacterial load in pulmonary drainage LN has not been observed. Nevertheless, this containment may not be sufficient to effectively prevent excretion of mycobacteria and horizontal transmission within a herd.

The role of the immunological status of vaccinated animals, especially the T-cell response, can be critical in terms of control or spread of the infection from the primary respiratory focus. Similarly, Hope *et al.* found few lesions at necropsy and smaller bacterial loads in LN in calves inoculated with *M. avium* and subsequently challenged with *M. bovis*[17]. The authors suggested that T-cell responses resulting from *M. avium* infection enhanced a protective secondary response after challenge with *M. bovis*.

After conducting cross-sectional analysis, we found positive correlations of the IFN-γ response to E/C with pathology severity, and more weakly with bacterial load. A similar result was obtained with the single ESAT-6-specific IFN-γ response compared with pathology scores in goats [30] and calves [33]. Furthermore, a positive correlation between the E/C-specific IFN-γ response and bacterial load was also previously described in goats [24] and cattle [34]. Moreover, after analyzing post-mortem data, clear differences in the pathological and bacteriological results were found within the vaccinated group. The IFN-γ responses of vaccinated goats with low and high post-mortem scores (VR and VNR, respectively) were compared. The significantly lower E/C-specific IFN-γ responses obtained in VR animals demonstrated the capacity of this immunological biomarker to predict the severity of the disease and, by default, to predict the vaccine outcome.

In terms of assessment of TB diagnostic tests, we have adapted the diagnostic tests routinely used in cattle in bovine TB eradication programs. We have also introduced new DIVA reagents to perform the IFN-γ assay. Sensitivities obtained in all tests were higher than those obtained in previous studies in naturally infected goats [20,23]. Notwithstanding this, the enhanced sensitivity described in these works when combining skin tests and IFN-γ tests was also confirmed in the present experimental trial.

The sensitivities of skin tests performed in vaccinated animals (100% and 90% for SIT and CIT, respectively), were higher than those obtained in another study carried out with goat herds naturally co-infected with TB and PTB (71% and 42.7%, respectively) [20]. In this work, the authors reported many animals with false-negative CIT results that showed higher skin-fold thickness to PPD-A than to PPD-B. It is important to note that the sensitivity of CIT in the present study was achieved using strict interpretation of the test. With the standard interpretation (ΔBov-ΔAv ≥ 3 mm), the sensitivity would be reduced to 60%.

By contrast, in our trial, only a slightly higher sensitivity was found when applying the SIT in comparison with the CIT. However, the results showed serious compromise of the specificity of the SIT in vaccinated animals prior to challenge (4 of 4 false-positive reactors), which completely disappeared when using the CIT. These results are in accordance with those found in another study performed in a PTB-vaccinated dairy goat herd free of TB [21]. Similarly, compromise of the specificity of the SIT in PTB-vaccinated deer has also been reported [35,36].

Compared with the SIT, the standard IFN-γ assay seemed to be more robust in terms of specificity. False-positive results were concentrated at the interval between weeks 12 and 14, and then disappeared at week 16 when there was not yet a response to *M. caprae* infection. After 12 wpv, the IFN-γ responses to PPD-A decreased faster than did those to PPD-B (see Figure 1). As a consequence, 60% of the animals were false-positive “bovine reactors” at week 14. More long-term trials must be performed to study the kinetics of the IFN-γ response to *Map* vaccination.

Encouragingly, the sensitivity obtained for the standard IFN-γ test after *M. caprae* challenge was very high considering that elevated whole-blood IFN-γ responses to PPD-A were previously observed in *Map*-vaccinated cattle [37]. In this sense, from weeks 18 to 28 (4–14 wpi), we only detected a masking due to PPD-A in two vaccinated animals. However, the high IFN-γ responses observed in our study (measured shortly after experimental infection) may decrease with the progression of natural TB infection under field conditions.

In the last decade, much effort has been focused on the development of novel antigens for bovine TB diagnosis that are more sensitive and specific than the avian and bovine tuberculins. This research has already resulted in the identification of several antigens, such as ESAT-6, CFP-10, and Rv3615c, that reduce cross-reactive immune responses to different mycobacterial infections or vaccinations in cattle [26,27,38] and goats [39,40]. In the present study, we showed the capacity of both E/C and Rv3615c to distinguish PTB*-*vaccinated and TB-infected goats. Furthermore, no differences in sensitivity were observed between the experimental groups. Importantly, when combining the positive results of E/C and Rv3615c IFN-γ assays, the sensitivity was identical to that obtained by the tuberculin-based IFN-γ assay, which is currently being employed as an ancillary test to the skin test in some eradication campaigns. A similar pattern was previously described in cattle experimentally infected with *M. bovis*, in which the sensitivity increased from 77.9% to 91% when considering the results of Rv3615c and E/C IFN-γ assay together [27].

By contrast, serological responses to the *Map* vaccine were moderate; only one and four vaccinated goats were seropositive to PTB at weeks 14 and 28, respectively. These data are not in agreement with the 50% positivity previously obtained in PTB-infected goats [41]. Interestingly, a boost effect on the *Map* ELISA due to CIT might be found at week 28 (2 weeks after tuberculin testing) when comparing the results with the test performed at week 14. The boost effect on the IgG response due to the skin test was previously described in *M. bovis*-infected cattle [34] and *M. caprae*-infected goats [24].

Vaccination against *Map* represents an important advance in controlling PTB and improving the economic balance of affected farms. Therefore, the pros and cons of its application must be exhaustively evaluated. An attractive speculation is that the partial protection to TB infection observed in some PTB-vaccinated animals could indirectly facilitate the control of TB, although long-term field studies are required to confirm this perspective. Moreover, we have demonstrated that to some degree, PTB vaccination interfered (after *M. caprae* infection) with the sensitivity of tuberculin-based TB diagnostic tests. However, beyond the first 2 weeks post-infection, only 5% false-negative results were obtained in the IFN-γ assay. In addition, these animals reacted positively in more than 80% of the remaining post-challenge tests. Thus, considering the collective basis on which TB tests are usually applied, it is unlikely that an infected herd could be undiagnosed upon extrapolation to a larger population. On the other hand, our results confirm that the interference with the specificity can be fully overcome by using defined DIVA reagents. Thus, developing and subsequently introducing these reagents into routine diagnostics could represent an improvement in both strategies: control of PTB by vaccination and control of TB by rapid detection of infected animals.

## Methods

### Experimental animals

The experiment was carried out in 20 Murciano-Granadina female goats between 2 and 3 months of age from a herd free of TB and PTB in the Region of Murcia (southwest Spain). Before the experiment, goats were submitted to standard tests for diagnosis of TB: the CIT and IFN-γ assay (Bovigam™; Prionics AG, Schlieren, Switzerland). Experimental animals were confirmed to be negative for both tests. In addition, all goats had negative results for PTB (Paratub.Serum-S; Institut Pourquier, Montpellier, France).

### Vaccination and infection

A group of 10 goats was subcutaneously inoculated with a single dose of 1 ml (2.5 mg/ml) of Silirum® (CZ Veterinaria, Porriño, Pontevedra, Spain), a commercial heat-inactivated and oil-adjuvanted *Map* vaccine. Another group of 10 goats was maintained as unvaccinated controls. After 14 weeks, all animals were housed and acclimatized in two experimental boxes (level 3 biocontainment) for 1 week prior to experimental infection.

The field strain of *M. caprae* SB0416 (http://www.mbovis.org) was used as the inoculum and was prepared as previously described [24]. Goats were anesthetized by intravenous administration of 4 to 6 mg/kg of propofol (Propofol Lipuro®) and 0.2 mg/kg of midazolam (Dormicum®), and subsequently inoculated with approximately 1.5 × 10^3^ cfu of *M. caprae* suspended in 0.5 ml of phosphate-buffered saline (PBS) by the endobronchial route [24].

All experimental procedures were approved by the Animal Welfare Committee of the Universitat Autònoma de Barcelona in agreement with the European Union Laws for protection of experimental animals.

### Sampling and clinical observations

Animals were observed twice daily at feeding time. Prior to vaccination and infection and every 2 weeks throughout the experiment, animals were weighed and their rectal temperature was taken and blood samples were collected from the jugular vein into heparinized blood tubes for immunological studies.

### Diagnostic tests

#### Skin tests

Skin tests were carried out in all goats at 26 wpv and 12 wpi. Four goats were also tested at 14 wpv (the remaining goats were not tested to minimize the potential effect of the intradermal tuberculin inoculation on the IFN-γ assay). Tests were performed by inoculating 0.1 ml (2500 IU) of bovine (PPD-B) and avian (PPD-A) tuberculins (Porriño, Pontevedra, Spain) on the left and the right side of the neck, respectively. The skin-fold thickness was recorded just before inoculation and after 72 h. Results were interpreted either by considering only the increase in thickness for PPD-B (as in a single intradermal test, SIT) or for both PPD-B and PPD-A (as in a comparative intradermal test, CIT). The results were read with the strict interpretation used in bovine TB eradication programs. Goats were considered positive to SIT if the increase in skin-fold thickness at the PPD-B site was ≥2 mm. For the CIT interpretation, goats were considered positive if the increase in skin-fold thickness at the PPD-B site was ≥2 mm and higher than the increase at the PPD-A site.

#### IFN-γ assay

Whole-blood cultures were performed every 2 weeks in 96-well cell culture plates. Heparinized blood (1 ml per well) was incubated for 24 h at 37°C and 5% CO_2_ with either PPD-B or PPD-A at a final concentration of 10 μg/ml. Phytohemagglutinin (PHA) (Sigma-Aldrich, Steinheim, Germany) was used as positive control at 10 μg/ml; PBS was used as a negative control. In addition, at 0, 2, 6, 10, and 14 wpi, the peptide cocktail ESAT-6/CFP-10 (E/C) and Rv3615c (Animal Health and Veterinary Laboratories Agency, Weybridge, UK) were used at a final concentration of 5 μg/ml. Plasma supernatants were collected after centrifugation and transferred to a 96-well plate. The IFN-γ enzyme-linked immunosorbent assay (ELISA) was performed according to the manufacturer’s instructions (Bovigam™). Optical density was measured at 450 nm (OD_450_) using a microplate reader (PowerWave XS; BioTek, Winooski, VT). Results were expressed as ΔOD_450_ (OD_450_ of antigen-stimulated sample minus OD_450_ of non-stimulated sample). A result was positive when ΔOD_450_ > 0.05, whereas in the case of the standard test (using PPD-A and PPD-B as stimuli), a result was positive when ΔOD_450_ of PPD-B > 0.05 and OD_450_ of PPD-B > OD_450_ of PPD-A.

#### Map IgG ELISA

Plasma samples were analyzed in duplicate for antibodies to *Map* with Paratub.Serum-S™ ELISA before vaccination (week 0), before infection (week 14), and before the end of the experiment (week 28). The assay was performed according to the manufacturer’s instructions. The ELISA reaction was also measured at OD_450_. The results were expressed as S/P (%), calculated as (mean of sample OD_450_ - mean of negative control OD_450_)/(mean of positive control OD_450_ - mean of negative control OD_450_) × 100. According to the criterion described by the manufacturer, a sample with an S/P of <45% was considered to be negative, of <55% and ≥45% to be doubtful, and of ≥55% to be positive.

### Post-mortem examination

All goats were euthanized at 14 wpi (28 wpv) with an overdose of sodium pentobarbital administered intravenously. They were immediately necropsied to assess the presence and volume of tuberculous lesions in the lungs and pulmonary LN.

#### Lungs

Ten percent-buffered formalin was poured into the trachea, which then was tied, and the lungs were subsequently immersed in a container with formalin. After fixation, the lungs were sliced at 4- to 5-mm intervals, and each slice was photographed. Gross lesions were analyzed using image analyzer software (ImageJ 1.43u; National Institutes of Health, USA). The volume of gross lesions in each slice was calculated by multiplying the affected area and slice thickness. The total volume of gross lesions was calculated by adding the partial volumes of gross lesions obtained for each slice.

#### Lymph nodes

The number of gross lesions and their diameter were recorded for each LN at the time of necropsy. Data were recorded by the same pathologist to ensure measurement consistency. LN pathology scoring was calculated by the approximated total volume of granulomas per sample, calculated using the sphere formula (4/3 × *π* × r^3^). After pathological evaluation, the whole LN was processed for bacterial culture to calculate the bacterial load.

### Bacterial count

Cranial and caudal mediastinal, tracheobronchial, and retropharyngeal LN were individually weighed and homogenized with 10 ml of sterile distilled water using a tissue homogenizer (Masticator; IUL Instruments, Barcelona, Catalonia, Spain). Homogenates were decontaminated with a final concentration of 0.35% w/v hexadecylpyridinium chloride [42] for 15 min with orbital shaking. Decontaminated homogenates were centrifuged at 2471 × *g* for 30 min, and pellets were resuspended in 10 ml of PBS containing 0.05% Tween 80. Aliquots of 0.1 ml of a 10-fold serial dilution of each homogenate were plated on Middlebrook 7H11 agar (BD Diagnostics, Sparks, MD). Plates were incubated at 37°C for 28 days, and colonies were then counted and the bacterial load (cfu/g) for each sample was calculated.

### Statistical analysis

Student’s unpaired two-sample *t*-test was used for comparisons between the groups in terms of differences in thickness increases in the CIT, S/P values of *Map* IgG ELISA, number of affected lung lobes, logarithm-transformed data of volumes of gross lesions (log_10_ mm^3^), and bacterial loads in the LN (log_10_ cfu/g). Correlations between pathology and bacterial burden were assessed using linear regression analysis, whereas the non-parametric Spearman rank test was used to analyze correlations between IFN-γ responses (ΔOD_450_) and post-mortem data. After cross-sectional analysis, IFN-γ responses and post-mortem parameters in the resulting groups were compared by applying the non-parametric Kruskal-Wallis test and Dunn’s *post hoc* multiple comparison test. The normality of the data and the homogeneity of variances between treatment groups were assessed using the Shapiro-Wilk and Levene tests, respectively. Statistical analysis of the data was performed using SPSS Statistical Package version 17.0 (IBM Inc., Chicago, IL).

## Competing interests

The co-author Eugenia Puentes belongs to CZ Veterinaria S.A., which produces Silirum® as a prophylactic vaccine against PTB. This does not alter the author’s adherence to the journal’s policy on sharing data. The rest of the authors declare that they have no competing interests.

## Authors’ contributions

BPV, RJ, and MD conceived and designed the experiments, analyzed the data, and drafted the manuscript. Regarding conduction of experiments, MD, SL-S, and MN performed the necropsy and evaluated the pathological records, and BPV and MM performed the immunological and bacteriological assays. JG, HMV, BVR, and EP contributed substantively to the scientific discussion of the results. All authors have read and approved the final manuscript.
